# COVID-19 in Piauí: initial scenario and perspectives for coping

**DOI:** 10.1590/0037-8682-0175-2020

**Published:** 2020-04-27

**Authors:** Francisca Miriane de Araújo Batista, Márcio Dênis Medeiros Mascarenhas, Natália Pereira Marinelli, Layana Pachêco de Araújo Albuquerque, Malvina Thais Pacheco Rodrigues, Marcelo Adriano da Cunha e Silva Vieira, Isaura Danielli Borges de Sousa

**Affiliations:** 1 Universidade Federal do Piauí, Centro de Inteligência em Agravos Tropicais Emergentes e Negligenciados, Teresina, PI, Brasil.; 2 Universidade Federal do Piauí, Centro de Inteligência em Agravos Tropicais Emergentes e Negligenciados, Programa de Pós-graduação em Saúde e Comunidade, Teresina, PI, Brasil.; 3 Universidade Federal do Piauí, Colégio Técnico de Teresina, Teresina, PI, Brasil.; 4 Universidade Federal do Piauí, Departamento de Enfermagem, Floriano, PI, Brasil.; 5 Universidade Federal do Piauí, Colégio Técnico de Teresina, Programa de Pós-graduação em Saúde e Comunidade, Teresina, PI, Brasil.; 6 Instituto de Doenças Tropicais Natan Portela, Teresina, PI, Brasil.; 7 Universidade Federal do Piauí, Departamento de Enfermagem, Floriano, PI, Brasil.

Dear Editor:

Coronavirus disease 2019 (COVID-19) has now resulted in a pandemic since the first case was reported in Wuhan, China in late 2019, despite global efforts to prevent its spread. According to an article published in April 2020, the first case of COVID-19 caused by the severe acute respiratory syndrome coronavirus 2 (SARS-CoV-2) in Brazil, was confirmed on February 26, 2020 by the Ministry of Health (MH). The patient was a 61-year-old man/woman from Sao Paulo, who recently arrived from Lombardy, northern Italy, where an outbreak occurred with a significant number of cases. Since the first case, the MH has disseminated technical recommendations and protocols to all states of the Federation^1^ ^-^ ^4^.

In Piauí, a state located in northeastern Brazil, the first suspected case of COVID-19 was reported on February 27, 2020. The first case was confirmed on March 19, 2020, and until the March 31, there were a total of 18 confirmed cases and 4 deaths due to COVID-19, with a fatality rate of 22.2%; two of these deaths occurred in Teresina, the capital of Piauí, where most of the confirmed cases are concentrated (16/18).

Teresina is the most populous city in Piauí (approximately 865 thousand inhabitants), with high connectivity by roads to other regions of the state and the only airport with direct domestic flights to several states in Brazil. In addition, it is the main site for highly complex care services in the developed Entre Rios region, located in the mid-north region of Piauí, with the remaining regions having fragile, fragmented, and vulnerable healthcare systems. This imposes an additional burden and challenge for health systems and economies in the countryside regions of the state in the face of the imminent scenario of SARS-CoV-2 dissemination.

Faced with the first suspected cases of COVID-19 in mid-February, the state government of Piauí developed the State Contingency Plan and instituted the Crisis Management Committee, both with the purpose of recommending and monitoring measures such as social-distancing and the suspension of collective activities, such as public events and school activities. On March 16, 2020, through Decree 18,884/2020, the government recommended the intensification of measures such as the suspension of collective activities or sporting, cultural, artistic, political, scientific, commercial and religious events; suspension of school classes; monitoring of suspicious cases; isolation for 14 days of travelers or those who had contact with confirmed cases; and the adoption of sanitary measures. 

In the meantime, the country published Decree 18,895, on March 19, 2020, declaring a state of public emergency, due to the serious public health crisis resulting from the COVID-19 pandemic, and its repercussions on public finances, thus enabling the enactment of exceptional measures to contain the spread of the disease in Brazil. In addition, Decrees 18,901 of March 19 and 18,902 of March 23, 2020 were published with measures to suspend economic activities due to the movement of people.

However, specific measures related to the health sector must be implemented, given that the state has 11 health regions and 227 intensive care unit (ICU) beds, of which 149 (65.6%) are concentrated in Teresina in the Entre Rios Region (Table 1), and 100% of the diagnoses by reverse transcriptase- C-reactive protein (RT-PCR) are made at the Central Public Health Laboratory, which is also located in the capital.

**TABLE 1:** Distribution of intensive care unit (ICU) beds, according to health regions-Piauí, Brazil, 2020.

Health regions	N	%
Entre Rios*	149	65.6
Planície Litorânea	43	18.9
Cocais	10	4.4
Vale do Canindé	10	4.4
Vale dos Rios Piauí and Itaueiras	10	4.4
Vale do Rio Guaribas	5	2.2
**Total**	**227**	**100.0**

Source: National Registry of Health Facilities. *Includes the Teresina city.

It is noteworthy that COVID-19 is not the only public health challenge today. Until March 2020, 441,224 probable cases (incidence coefficient of 209.9 cases per 100,000 inhabitants) of dengue were reported in the country. The Northeast Region had an incidence of 49.5 cases/100,000 inhabitants, with an increasing trend, but still within the expected level (endemic channel). Simultaneously, 2,184 suspected cases of measles were reported, of which 338 (15.5%) were confirmed (incidence rate of 0.8/100,000 inhabitants). In this difficult epidemiological scenario, there is a clear expectation of witnessing a measles, dengue, and COVID-19 syndemia, among other conditions that afflict the Brazilian population^5^ ^-^ ^7^.

In this context, the healthcare system in Piauí, like other regions of Brazil and the world, is not prepared to face this pandemic that is growing at alarming rates across the world. The rapid spread of the epidemic is partly due to the delay in testing suspected cases, providing results, and isolating patients, in addition to the failure to protect healthcare professionals, resulting in dissemination from healthcare providers^8^.

Therefore, the government must immediately pay attention to the need to expand healthcare infrastructure to combat COVID-19. It is necessary for the population and healthcare professionals to be guided to adopt the preventive measures recommended by experts and public health officials. The population must perform hand hygiene and avoid closed environments and avoid contact with people from regions with COVID-19 outbreaks. Healthcare professionals should use protective glasses or face shields, surgical/N95 masks, disposable aprons, and procedure gloves and always wash their hands, especially when aiding suspected or confirmed cases of COVID-19. In this scenario, there is a need for governments to establish their commitment to offering the entire population the mitigation measures recommended to overcome this crisis^9^. In addition, the present scenario highlights the need to strengthen the Brazilian Unified Health System as well as the need for massive investment in research, technology, and innovation to effectively combat public health emergencies in our country.
